# Chronic diseases and health conditions in adolescents: Sex inequalities

**DOI:** 10.1590/1980-549720230009.2

**Published:** 2023-01-09

**Authors:** Vivian Castro Lemos, Marilisa Berti de Azevedo Barros, Margareth Guimarães Lima

**Affiliations:** I Universidade Estadual de Campinas – Campinas (SP), Brasil.

**Keywords:** Adolescent, Chronic disease, Health status, Demography, Adolescentes, Doenças crônicas, Condições de saúde, Inquéritos populacionais

## Abstract

**Objective:**

To estimate the prevalence of chronic diseases and health conditions in adolescents from Campinas (São Paulo), investigating sex differences according to age group.

**Methods:**

This population-based study analyzed data from the ISACamp 2014/15 health survey, with a total of 1,022 adolescents interviewed. The interviewees consisted of 517 boys and 505 girls; 492 of them in the ten to 14 age group and 530 in the 15 to 19 age group. We verified the associations using the χ^2^ test with Rao Scott adjustment and estimated prevalence ratios (PR) with multiple Poisson regression adjusted for age. Analyses were also stratified by age group.

**Results:**

Respiratory diseases, such as rhinitis (25.3%), sinusitis (15.7%), and asthma (10.9%), were the most prevalent among adolescents. Health complaints were high, especially headaches (39.5%), emotional conditions (34.5%), allergies (27.5%), and back pain (21.3%). More than 22.0% of adolescents reported having three or more health conditions. Girls declared a higher number of health conditions (three or more) than boys (PR=2.27).

**Conclusion:**

The study showed that adolescents presented a significant number of health conditions, particularly regarding complaints, indicating the need for clinical care and public policies aimed at controlling and preventing these diseases in this age group.

## RESUMO

**Objetivo:**

Estimar as prevalências de doenças crônicas e problemas de saúde em adolescentes de Campinas (SP), observando as diferenças entre os sexos, por faixa etária.

**Métodos:**

Estudo em base populacional, com dados do inquérito de saúde ISACamp de 2014/15, cujas entrevistas com adolescentes totalizaram 1.022. Desses entrevistados, 517 eram meninos e 505, meninas; 492 encontravam-se na faixa de dez a 14 anos e 530 tinham entre 15 e 19 anos. As associações foram verificadas por meio do teste de χ² com ajuste de Rao Scott, e as razões de prevalência (RP) foram estimadas por meio de regressão múltipla de Poisson ajustadas por idade. Também foram feitas análises estratificadas por faixa etária.

**Resultados:**

As doenças respiratórias foram as mais prevalentes nos adolescentes como rinite (25,3%), sinusite (15,7%) e asma (10,9%). As queixas de saúde apresentaram-se elevadas, destacando-se as dores de cabeça (39,5%), problemas emocionais (34,5%), alergias (27,5%) e dores nas costas (21,3%). O número de adolescentes que apontaram ter três ou mais problemas de saúde mostrou-se acima de 22,0%. As meninas referiram maior número de problemas de saúde (três ou mais) do que os meninos (RP=2,27).

**Conclusão:**

O estudo demonstrou que os adolescentes apresentaram número expressivo de problemas de saúde, principalmente em relação às queixas, sinalizando que são necessários cuidados clínicos e políticas públicas direcionadas para o controle e prevenção desses agravos nesta faixa etária.

## INTRODUCTION

Adolescence is a life stage filled with changes in physical, cognitive, and emotional status and behaviors. Some factors involved in the process of transition to adulthood concern the consolidation of self-care and body notions. Thus, family spaces and school environments, as well as specific public policies, influence this journey^1^.

Health policies aimed at adolescents have been on the rise worldwide, despite the considerable challenges they face, among which the inattention of society for assuming this population has fewer care needs compared to other age groups, leading to negligence regarding complaints and chronic diseases in adolescents^2–6^.

Recent data from the World Health Organization identified external causes, injuries, psychiatric disorders, maternal and contraception complications, and infectious diseases as the main causes of death among young people^4^. Preventable and external causes differed between sexes and regions, although road traffic accidents, infections, drowning, and depressive disorders were similar between high- and low-income countries^7^. After analyzing mortality trends between ten and 24 years, from 1990 to 2019, using data from the Global Burden of Disease research, a Brazilian study reported that the main causes of death were external. Among women, they were: road injuries, followed by interpersonal violence, maternal deaths, and suicide. For men, interpersonal violence was the first cause of death, followed by road injuries, suicide, and drowning^5^.

Chronic noncommunicable diseases can affect individuals throughout their lives and are multifaceted, ranging from genetics to external factors^8^ and lifestyle^9^. Health risk behaviors, such as smoking, alcohol consumption, unhealthy eating, and physical inactivity, tend to start at an early age and strongly influence the development of diseases, health conditions, and multimorbidities^2^. Few population-based studies describe the prevalence of chronic diseases and other health complaints in this age group^3,10,11^.

Headaches and musculoskeletal pain are usually significant in adolescence^3^, possibly due to or associated with emotional conditions^12,13^. These emotional disorders can have multifactorial and pathological determinants^14^, affecting social and professional relationships in future ages. Pain in this age group, especially musculoskeletal ones, might result from physical development — inherent in growth —, exhausting efforts, or physical inactivity^12^.

Adult women tend to report worse health conditions, while men die earlier, indicating gender paradoxes, as well as biological and self-care differences. Women usually declare more emotional conditions, the presence of diseases and other complaints, and access health services more often. On the other hand, men are prone to expose themselves to risk conditions, underestimate their symptoms, and do not recognize their own health conditions, leading to early deaths^15,16^.

Brazilian studies addressing comprehensive and aggregated perspectives on health status and diseases in adolescents, according to sex, are scarce. Although some studies focus on adolescents in Brazil, such as the Study of Cardiovascular Risks in Adolescents (*Estudo de Riscos Cardiovasculares em Adolescentes* — ERICA) and the National Survey of School Health (*Pesquisa Nacional de Saúde em Escolares* — PeNSE), few analyses include the prevalence of a group of various chronic diseases and health conditions. Understanding gender inequalities in health and knowing whether they have been recurrent since adolescence is important for developing more efficient health actions and specific public policies^17^.

Therefore, this study aimed to present the prevalence of chronic diseases and the main health conditions reported by adolescents from Campinas, investigating the association according to sex, with stratifications by age group.

## METHODS

This population-based cross-sectional study used data from the Campinas Health Survey, carried out in 2014/2015.

A total of 3,021 individuals were interviewed, with a random selection of 2,898, 950, and 3,326 households to find adolescent, adult, and older adult respondents, respectively. All residents of the age group selected from each household were interviewed.

The sampling plan for adolescents considered both sexes and the age range of ten to 19 years. The sample selection was estimated to be a thousand people, considering a 50% proportion, 0.5 variability, 95% confidence coefficient, and a design effect of 2. Initially, 70 census tracts were randomly selected, listing 14 of them in each of the municipal health districts of Campinas (east, northwest, north, southwest, and south), followed by the selection of households.

Information was gathered by a precoded questionnaire administered by trained interviewers. All interviewees signed the Informed Consent Form (ICF). Survey data were collected in a tablet and submitted for consistency evaluation.

The questions asked to obtain information about diseases were: “Has any doctor ever diagnosed you with…”; and for health conditions: “Do you have/usually have…”. In this scenario, the group of dependent variables (outcomes) used was:

- Chronic morbidities (self-reported diagnosis of morbidities): rhinitis, sinusitis, asthma, high cholesterol, spinal disease/condition, bronchitis/chronic obstructive pulmonary disease (COPD), and hypertension.
- Health conditions (complaints or symptoms): migraine/headache, emotional/mental condition, allergies (except rhinitis, sinusitis, and asthma), back pain, dizziness/vertigo, insomnia, and urinary tract infection/cystitis.
- The variable number of chronic diseases was constructed by adding all other diseases mentioned in the questionnaire, including — in addition to the seven listed above — the following: diabetes, angina, cardiac arrhythmia, infarction, other heart diseases, cancer, osteoporosis, other lung diseases, tendonitis/repetitive strain injury (RSI), varicose veins, stroke, other circulatory diseases.
- The variable number of health conditions was elaborated by calculating all events cited in the interview, that is, the seven mentioned above, as well as urinary incontinence and other health conditions.

The categorizations defined were: none, one, two, and three or more morbidities, which characterized the multimorbidities.

All morbidities and health conditions collected in the 2014/2015 survey and included in the checklist were investigated. However, only the most prevalent ones were analyzed and described (Tables 1 and 2). Sex analyses stratified by age group were also performed when the overall sex associations were significant (Table 3).

**Table 1.** Prevalence of chronic diseases and health conditions in adolescents. Campinas (São Paulo), ISACamp 2014/15.

Variables	N*	**Prevalence (%)** **(95%CI)** ^**†**^	**Prevalence (%) by age group** **(95%CI)** ^**†**^	
			10 to 14	15 to 19
Chronic diseases				
Rhinitis	42,719	25.3 (21.7–29.2)	24.6 (20.1–29.9)	25.9 (21.8–30.5)
Sinusitis	26,608	15.7 (12.91–19.1)	13.9 (10.7–18.0)	17.4 (14.0–21.5)
Asthma	18,460	10.9 (9.1–13.1)	10.7 (8.2–13.8)	11.2 (8.7–14.2)
High cholesterol	6,931	4.1 (2.9–5.8)	5.9 (4.1–8.5)^‡^	2.4 (1.3–4.3)^‡^
Spinal disease/condition	6,018	3.6 (2.5–5.1)	2.1 (1.1–4.1)^‡^	4.9 (3.2–7.6)^‡^
Bronchitis/COPD	4,665	2.8 (1.8–4.1)	2.1 (1.2–3.7)	3.3 (1.9–5.9)
Hypertension	2,451	1.5 (0.8–2.6)	1.3 (0.5–3.0)	1.6 (0.7–3.5)
Number of chronic diseases				
0	91,490	54.1 (49.4–58.8)	55.4 (49.7–61.0)	52.9 (46.9–58.9)
1	46,320	27.4 (23.9–31.2)	28.4 (24.1–33.2)	26.4 (21.7–31.8)
2	21,452	12.7 (10.2–15.6)	11.6 (8.4–15.8)	13.7 (10.6–17.5)
3+	9,788	5.8 (4.0–8.3)	4.5 (3.1–6.7)	6.9 (4.4–10.8)
Health conditions				
Migraine/headache	66,741	39.5 (34.9–44.3)	39.0 (33.5–44.7)	40.0 (34.8–45.4)
Emotional/mental condition	58,373	34.5 (28.0–41.6)	32.9 (25.5–41.3)	36.0 (29.5–43.2)
Allergies	46,455	27.5 (24.3–30.9)	26.9 (22.9–31.2)	28.1 (23.7–32.9)
Back pain	35,923	21.3 (18.0–25.0)	15.7 (12.6–19.4)^‡^	26.4 (21.5–32.0)^‡^
Dizziness/vertigo	21,740	12.9 (10.6–15.5)	10.0 (7.4–13.5)^‡^	15.5 (12.3–19.4)^‡^
Insomnia	19,711	11.7 (9.4–14.4)	9.4 (7.1–12.5)^‡^	13.7 (10.9–17.2)^‡^
UT infection/cystitis	7,150	4.2 (3.0–5.9)	3.1 (1.8–5.3)	5.3 (3.6–7.7)
Number of health conditions				
0	46,218	27.3 (22.4–32.9)	29.2 (23.6–35.7)	25.6 (20.1–31.9)
1	48,822	28.9 (26.4–31.5)	31.0 (27.5–34.7)	26.9 (23.5–30.6)
2	36,363	21.5 (18.6–24.7)	21.0 (17.2–25.5)	21.9 (18.4–25.9)
3+	38,155	22.6 (18.6–26.5)	18.7 (14.4–23.9)^‡^	25.6 (21.6–30.1)^‡^

COPD: chronic obstructive pulmonary disease; UT: urinary tract *Total of adolescents with health conditions in Campinas. 2014/2015 population estimate; ^†^95% confidence interval; ^‡^p-value<0.05. Source: Tabnet/DATASUS/Campinas.

**Table 2.** Prevalence and prevalence ratios of the association between sex, chronic diseases, and health conditions in adolescents. Campinas (São Paulo), ISACamp 2014/15.

Variables	Male (1) (%)	Female (2) (%)	PR* (2/1) ^**†**^
Chronic diseases			
Rhinitis	24.1	26.5	1.10 (0.89–1.36)
Sinusitis	14.8	16.7	1.12 (0.85–1.50)
Asthma	13.1	8.6	**0.65 (0.47–0.90)** ^‡^
High cholesterol	2.8	5.5	**2.01 (1.11–3.64)** ^‡^
Spinal disease/condition	2.5	4.7	1.87 (0.95–3.70)
Bronchitis/COPD	3.1	2.4	0.77 (0.38–1.55)
Hypertension	1.6	1.3	0.85 (0.25–2.82)
Number of chronic diseases			
0	54.6	53.6	1
1	28.6	26.2	0.90 (0.74–1.09)
2	11.7	13.7	1.14 (0.84–1.56)
3+	5.1	6.5	1.25 (0.84–1.88)
Health conditions			
Migraine/headache	**30.0** ^‡^	49.3	**1.64 (1.35–2.00)** ^‡^
Emotional/mental condition	29.2	40.1	**1.37 (1.15–1.63)** ^‡^
Allergies	26.7	28.3	1.06 (0.86–1.30)
Back pain	15.4	27.3	**1.76 (1.30–2.38)** ^‡^
Dizziness/vertigo	9.5	16.3	**1.70 (1.25–2.30)** ^‡^
Insomnia	10.0	13.4	1.33 (0.90–1.96)
UT infection/cystitis	0.8	7.8	**9.77 (3.45–27.65)** ^‡^
Number of health conditions			
0	32.3	22.3	1
1	32.7	24.9	**0.76 (0.62–0.93)** ^‡^
2	21.3	21.7	1.02 (0.77–1.35)
3+	13.7	31.2	**2.27 (1.65–3.12)** ^‡^

COPD: chronic obstructive pulmonary disease; UT: urinary tract. *prevalence ratios adjusted for age; ^†^reference: males; ^‡^significant differences.

**Table 3.** Prevalence ratios of the association between sex, chronic diseases, and health conditions according to age group. Campinas (São Paulo), ISACamp 2014/15.

Variables	PR (95%CI)	
	10 to 14 years*	15 to 19 years*
Asthma	**0.57 (0.34–0.97)** ^†^	0.73 (0.44–1.19)
Cholesterol	1.44 (0.68–3.07)	**5.82 (1.11–30.31)** ^†^
Migraine/headache	**1.38 (1.06–1.78)** ^†^	**1.95 (1.50–2.54)** ^†^
Emotional/mental condition	**1.41 (1.03–1.91)** ^†^	**1.34 (1.10–1.62)** ^†^
Back pain	**1.62 (1.01–2.59)** ^†^	**1.83 (1.31–2.55)** ^†^
Dizziness/vertigo	1.28 (0.76–2.15)	**2.02 (1.36–3.01)** ^†^

PR: prevalence ratio; CI: confidence interval. *Reference: males; ^†^significant differences

The independent variables of the study were sex (female and male) and age group (10–14 and 15–19 years). The sex analyses used continuous age for the proper adjustments.

We calculated prevalence estimates and 95% confidence intervals (95%CI) and tested associations using Pearson’s χ^2^ test with Rao Scott adjustment for more robust corrections in complex samples. Prevalence ratios (PR) were estimated by applying multiple Poisson regression models with robust variance. Data analysis considered weightings and characteristics related to sample design using the Stata 15.0 Software.

The Ethics Committee of the School of Medical Sciences at Unicamp approved the ISACamp 2014/2015 survey, under Opinion no. 409,714/2013, and the current project, under no. 5,283,905/2022, according to Resolution no. 466 from December 12, 2012.

## RESULTS

A total of 1,022 adolescents with a mean age of 14.5 years (95%CI 14.3–14.7) were interviewed. Among them, 50.9% (95%CI 47.4–54.2) were male (n=517) and 49.1% (95%CI 45.7–52.5) were female (n=505), 492 were aged 10 to 14 years and 530 were aged 15 to 19 years.

The most reported chronic diseases were those related to respiratory conditions, with a prevalence of 25.3% for rhinitis, 15.7% for sinusitis, and 10.9% for asthma. Other diseases presented percentages below 5% (high cholesterol, spinal conditions, bronchitis, and hypertension). The prevalence of high cholesterol was greater in girls (PR=2.01), while asthma was higher among boys (PR=0.65). The overall prevalence of health conditions in this age group was quite high, especially headaches (39.5%) and emotional conditions (34.5%). Allergies (27.5%) and back pain (21.3%) had percentages above 20.0%. Dizziness and insomnia were reported by over 10.0% of the adolescents, and two out of ten of them declared having three or more health conditions. Although little reported (4.2%), cystitis was a predominantly female symptom. Five of the seven health conditions analyzed showed statistically significant differences between sexes, with migraine/headache (PR=1.64), emotional/mental condition (PR=1.37), back pain (PR=1.76), dizziness or vertigo (PR=1.70), and urinary tract infection (PR=9.77) standing out among girls, who also reported three or more health conditions over twice as often as boys (PR=2.27) (Tables 1 and 2).

The prevalence of high cholesterol and dizziness/vertigo was greater in girls, but the differences were found only in the age range of 15 to 19 years. On the other hand, the asthma prevalence was lower in girls, with differences only among the youngest (ten to 14 years) (Table 3).

## DISCUSSION

Adolescents from Campinas proved to have diagnoses of chronic diseases, particularly those related to the respiratory system, with prevalence ranging from 10.9% (asthma) to 25.3% (rhinitis). Health complaints and symptoms, as well as the clustering of health conditions, presented high frequencies, especially among girls, indicating that adolescents are getting sick and that females, since their youth, tend to express their health difficulties and needs more often.

Data from the National Household Sample Survey (*Pesquisa Nacional por Amostra de Domicílios* — PNAD) 2008 revealed at least one chronic disease in 11.2% of adolescents in Brazil^3,18^, a number that has been rising alarmingly, with respiratory diseases being the most frequent^19^. Morbidities such as rhinitis and sinusitis regularly affect adolescents worldwide^3,19^.

Information from ISACamp 2008 showed a 7.6% prevalence of asthma^3^. Among students, PeNSE showed a downward trend in the prevalence of asthma symptoms, such as wheezing, when comparing 2012 (25.2%) and 2015 (23.5%), with girls presenting the highest incidence of these symptoms (26.7%). Regarding medical diagnosis, ERICA 2013/2014 indicated 8.7% of asthma diagnoses between the ages of 12 and 17 years, a value higher in girls^20^.

Results of this study pointed to asthma as the third most prevalent disease, reinforcing national and international findings of common diseases in the analyzed life stage^21–23^. Self-reported rhinitis and sinusitis were more predominant in girls, contrary to the asthma trend, which was mainly present in boys. These findings corroborate those of an Investigation carried out in educational institutions in Northeastern Brazil, whose results revealed a greater association of asthma in boys^24^. Evidence indicates that asthma prevalence is higher in boys during childhood but tends to increase in girls throughout the hormonal changes of adolescence^25^. These diseases, especially asthma and rhinitis, are associated and may unfold into symptoms such as insomnia and infections, leading to limitations and difficulties in adulthood.

Population data on abnormal lipid profiles in adolescents are scarce in the country. However, results from laboratory tests in adolescents collected by ERICA 2013/2014 showed a greater preponderance of high values in girls (24.9%) and a total mean of 20.1%, a figure five times higher than that found by this study (4.1%), representing important cardiovascular risks, even in children and adolescents. An evaluation of lipid profiles of children and adolescents in primary health care units of Campinas, conducted between 2008 and 2015, indicated increased frequencies of around 30.0% in cholesterol and 40.0% in triglycerides^26^. Given the low number of complications and mortality caused by dyslipidemias in adolescents, routine tests are unusual in this population, which can suggest underreporting and a lack of knowledge of the problem. The result found in Brazilian adults points to a 12.5% prevalence of dyslipidemia, according to the National Health Survey^27,28^. This scenario prompts reflections on the need to educate the population on how to identify dyslipidemias early in children, adolescents and young adults, which does not seem to be the case, considering that the individual’s knowledge of diagnosis is less than the screening performed by laboratory tests.

Discussing chronic diseases in adolescents is complex, as the questions and indicators used in research do not coincide, and some do not include adolescents aged ten to 19 years, categorizing the participants into narrower age ranges or school ages. In addition, the methodologies of the questionnaires differ.

The analysis of complaints and symptoms shows even more distinct patterns, in which migraine or headache presented a high prevalence in 2015 (39.5%), with a much higher trend in girls (PR=1.64). In 2016, 40.8% of adolescents from a Northeastern cohort complained of headaches, a factor that negatively affects the quality of life and may relate to high screen time and emotional conditions, besides being a consequence of low sleep quality^29^, also reported by 11.7% of adolescents from Campinas who complained of insomnia, in our study.

Emotional conditions were significant in this Investigation (34.5%), with a high prevalence ratio in girls. The ERICA study used an assessment instrument for common mental disorders (CMD), the General Health Questionnaire (GHQ-12), revealing a 30.0% prevalence, similar to our findings, although estimated by different methodologies^11^. Brazil still needs to advance in mental health care for children and adolescents, who, although minimally supported by the Child and Adolescent Statute (*Estatuto da Criança e do Adolescente* — ECA) and the Child and Adolescent Psychosocial Care Centers (*Centros de Atenção Psicossociais Infanto-juvenis* — CAPSij), still do not seem to be sufficiently assisted^1,30^. Additionally, studies tend to evaluate psychiatric diagnoses without connecting them to self-reported sensations, feelings, and suffering, which could improve care in this age group, with early interventions^31–33^. Some works indicate important prevalence rates of emotional conditions in adolescence, indicating that these disorders can negatively influence physical and psychological development and persist in adulthood, leading to their worsening^34,35^.

When assessing back pain, this study obtained very similar findings (21.3%) to those of a São Paulo survey of the same year (22.4%), which evaluated young people aged 15 to 19 years. As this complaint has a multifactorial nature and various origins, back pain is associated with many work leaves, resulting in severe limitations throughout life, which can worsen without proper treatment and care^36^. In the country, the prevalence in adults ranged from 30.0 to more than 60.0%^37,38^. International studies estimate reports of 37.0% back pain among the population aged nine to 17 years^37–39^. Despite its regular appearance, this condition receives little attention, especially during adolescence, often identified as inadequate body postures and/or lack and excess activities^40,41^.

Many young people present dizziness and/or vertigo symptoms, that is, a sensation of movement, being off-balance, unsteadiness, and even falls, which can relate to various diseases and other health complaints such as headaches^42^. Quite prevalent in the female population, it tends to increase with advancing age^42^. In the present study, 12.9% of adolescents reported dizziness and/or vertigo. In 2011, a study conducted in Minas Gerais found that, among individuals who declared having some kind of health condition, 6.7% stated having dizziness symptoms^43^, but several authors indicate prevalence rates with wide ranges — from 11.0 to 42.0% —, influenced by subjectivity and underestimation of this perception^42–47^.

The association between cystitis and sex (PR=9.77) was strong, with higher occurrence in girls, findings consistent with the evidence in the literature, as this population is more susceptible to typical urinary tract symptoms, and the condition may happen in parallel to vaginitis and sexually transmitted infections (STI), which should be satisfactorily investigated^48,49^.

A large number of these conditions can be associated with modifiable risk factors, such as inadequate diets, alcohol and tobacco use, as well a sedentary lifestyle, physical inactivity, and overweight^10^. Implementing a healthy lifestyle to reduce negative effects in the future should be crucial even in this transition between childhood and adulthood.

A cohort study conducted in Pelotas (Rio Grande do Sul) identified a relationship between tobacco use and mental health conditions in adolescents aged 15 years, diagnosed with the Strengths and Difficulties Questionnaire (SDQ) instrument^50^. PeNSE 2015 revealed an increased use of other tobacco products in Brazilian adolescents, especially waterpipe, demanding the monitoring of these risk factors in this population^10^. Physical activity and sports have also been the object of national and international recommendations related to the prevention and control of chronic diseases^51^, as well as the reduction of complaints of pain and emotional conditions^52^.

PeNSE and ERICA results clearly showed males being more active in leisure and commuting to school than females, even though the percentage in the age group as a whole is low^53,54^. Since childhood, boys tend to be urged to play sports and participate in active plays, while girls are encouraged not to go out without company, play at homes, and in a more sedentary way^55^.

When estimating the health conditions or symptoms reported, male adolescents presented lower percentages than female ones. This evidence shows that, even in adolescence, the male population tends to have more difficulties accepting their diseases, particularly when disclosing this information during medical visits or when requested on different occasions^15,56^.

Aside from physiological differences, since adolescence, the approach to masculinity is often linked to virility, power, and risk exposure, as well as difficult relationships with these individuals in healthcare facilities^57^. Launched in 2009, the National Policy for Comprehensive Male Health Care (*Política Nacional de Atenção Integral à Saúde do Homem* — PNAISH) proposed to work with primary health care units to qualify care for this population, favoring care and benefits for adult men^58^. Therefore, making health education more flexible when these individuals are still boys might favor better health outcomes later^59^. The higher frequency of several health events reported in females may also relate to their wide demand for health services, while mortality rates show greater numbers in males, involving associated risk behaviors, such as alcohol abuse, tobacco use, and increased exposure to violent and vulnerable situations^15^. Understanding health and disease aspects in the domain of sex differences goes far beyond biological and genetic particularities between men and women, spanning social inequalities and various contexts of male cultural behaviors and reverberating in the fact that they seem to be healthier by not identifying conditions and/or diseases. On the other hand, women are constantly encouraged to take care of their health, in addition to being exposed to reinforcement in body awareness and demonstration of their weaknesses^57,60^.

Considering the importance of sex differences in health, which begin in adolescence, fostering debates is necessary to deepen this theme in the various health, promotion, and protection policies for adolescents. Nonetheless, some publications alert to the issue of teenage pregnancy and potential risks, in addition to mentioning and giving attention to women’s health and to the importance of reproductive and sexual education aimed at adolescents^61–63^.

Among the limitations of the work, we underline the use of information reported by adolescents on chronic diseases. This information may be related to memory bias and lack of knowledge of the problem due to the low access and use of services by the younger and most socially vulnerable population, and thus, the prevalence of reports of diagnosis of the health conditions described could be underestimated.

Notably, the scenario presented in this study is prior to the COVID-19 pandemic and records data for future comparisons on the prevalence of health conditions among adolescents in an important metropolis in the state of São Paulo, Brazil.

Chronic diseases, especially those involving the respiratory tract and health complaints, were very prevalent among adolescents from Campinas. The high frequency of reported complaints and symptoms may be linked to underreporting of diagnoses, exposure to stressful situations, and unhealthy lifestyles. Efforts to take strategic actions that consider heterogeneity in different dimensions of the health-disease process among adolescent boys and girls are essential since some morbidities are very significant in this population. Moreover, strengthening health promotion programs and policies is crucial to support and leverage health protection factors among adolescents.
